# Fine-scale spatial segregation in a pelagic seabird driven by differential use of tidewater glacier fronts

**DOI:** 10.1038/s41598-021-01404-1

**Published:** 2021-11-11

**Authors:** Philip Bertrand, Joël Bêty, Nigel G. Yoccoz, Marie-Josée Fortin, Hallvard Strøm, Harald Steen, Jack Kohler, Stephanie M. Harris, Samantha C. Patrick, Olivier Chastel, P. Blévin, Haakon Hop, Geir Moholdt, Joséphine Maton, Sébastien Descamps

**Affiliations:** 1 Département de biologie, chimie et géographie & Centre d’études nordiques, Université du Québec à Rimouski, Rimouski, G5L 3A1 Canada; 2 Norwegian Polar Institute, Fram Centre, Tromsø, 9296 Norway; 3 Department of Arctic and Marine Biology, UiT The Arctic University of Norway, Tromsø, 9037 Norway; 4 Department of Ecology and Evolutionary Biology, University of Toronto, Toronto, M5S 3B2 Canada; 5 Cornell Lab of Ornithology, Cornell University, 159 Sapsucker Woods Road, Ithaca, NY 14850 USA; 6 School of Environmental Sciences, University of Liverpool, Liverpool, L69 3GP UK; 7 Centre d’études biologiques de Chizé (CEBC), UMR 7372, CNRS & Université de La Rochelle, Villiers-en-Bois, 79360 France; 8 Akvaplan-niva, Fram Centre, Tromsø, 9296 Norway

**Keywords:** Behavioural ecology, Biogeography

## Abstract

In colonially breeding marine predators, individual movements and colonial segregation are influenced by seascape characteristics. Tidewater glacier fronts are important features of the Arctic seascape and are often described as foraging hotspots. Albeit their documented importance for wildlife, little is known about their structuring effect on Arctic predator movements and space use. In this study, we tested the hypothesis that tidewater glacier fronts can influence marine bird foraging patterns and drive spatial segregation among adjacent colonies. We analysed movements of black-legged kittiwakes (*Rissa tridactyla*) in a glacial fjord by tracking breeding individuals from five colonies. Although breeding kittiwakes were observed to travel up to ca. 280 km from the colony, individuals were more likely to use glacier fronts located closer to their colony and rarely used glacier fronts located farther away than 18 km. Such variation in the use of glacier fronts created fine-scale spatial segregation among the four closest (ca. 7 km distance on average) kittiwake colonies. Overall, our results support the hypothesis that spatially predictable foraging patches like glacier fronts can have strong structuring effects on predator movements and can modulate the magnitude of intercolonial spatial segregation in central-place foragers.

## Introduction

Identification of key factors driving animal distribution patterns and movements is a fundamental goal in ecology^1,2^. In various species, individuals must return to a central base between foraging bouts, greatly shaping their spatial ecology^3–5^. Optimal foraging models assume that central‐place foragers, such as colonial-nesting species, are adapted to maximize their rate of net energy gain per unit of time and that the costs of foraging increase with increasing distance from the colony^6,7^. If foragers do not exhibit territorial defence and if prey are abundant and uniformly and/or unpredictably distributed, then the theory predicts that the density of foragers should decrease with increasing distance from the colony. Under such conditions, foraging areas of neighbouring colonies could overlap when separated by less than the distance covered by their respective foraging ranges. However, segregated foraging grounds of neighbouring colonies appear widespread among colonial central‐place foragers like eusocial insects^8^, bats^9^, pinnipeds^10^, and seabirds^11^.

Several factors could promote spatial segregation between neighbouring colonies in seabirds, including intraspecific competition^12,13^, distribution of predators^14^, individual specialization in space use^11^, and associated cultural effects^5,13,15^. Prey distribution around colonies is another potential driver of their spatial segregation^16–18^. Since optimal foraging models predict that individuals should mainly exploit patches closer to their colony to maximize their net energy gain^6,7^, prey distribution and physical structures of the seascape that lead to their aggregations could have an important effect on how animals distribute themselves within the shared foraging ranges^11,17,18^. The patchiness of prey (i.e., patch size and distribution) and its associated predictability (i.e., spatial and temporal) appear as important drivers of the spatial structuring of seabird movements^19–21^. These factors could thus modulate the level of segregation between neighbouring colonies^11,16,17^.

Tidewater glaciers fronts are known to be important foraging hotspots for seabirds and other marine predators^22–24^. They have recently gained increasing attention due to their potential to alleviate the short-term negative effect of ice-associated habitat loss on Arctic wildlife^25,26^. Driven by the meltwater glacier discharge at the fronts, zooplankton are entrained and transported by the buoyant subglacial plumes at the ice-sea interface, making prey readily available at the surface for predators^22,27–29^. Although their relative profitability can vary over time^30–34^, glacier fronts may nonetheless represent spatially predictable foraging habitats for breeding colonial seabirds^30,31,33^ due to their geographically restricted and relatively fixed location. Despite their documented importance as foraging hotspots, little is known about their structuring effect on predator distribution patterns and movements.

Focusing on Arctic breeding black-legged kittiwakes (*Rissa tridactyla*), our study aims at testing the hypothesis that tidewater glacier fronts represent high value patches that modulate seabird foraging patterns and generate fine-scale spatial segregation among neighbouring colonies. Kittiwakes are surface-feeders, central place foragers during the breeding season, and are commonly observed at tidewater glacier fronts^22,30,31^. We predicted that kittiwakes breeding within a glacial fjord (Kongsfjorden, Svalbard) in the Arctic should predominantly use glacier fronts located closer to their colony. Then, while controlling for differences in colony size and distance separating colonies^11,13^, we further predicted that differential use of glacier fronts promotes a fine-scale spatial segregation (i.e., less overlap than what would be expected from distance alone) among neighbouring colonies.

## Methods

### Study system

**Figure 1:**
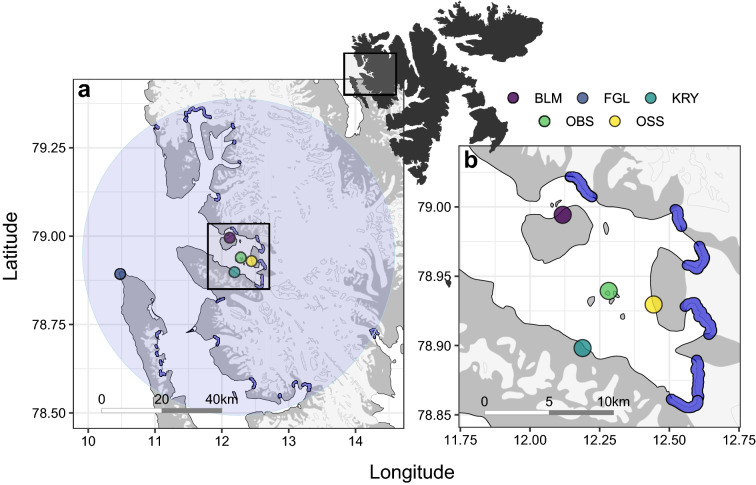
Svalbard archipelago (top; land in black), (**a**) the 50 km radius area corresponding to the regional scale, and (**b**) the Kongsfjorden area corresponding to the fjord scale. Kittiwake breeding colonies (circles: BLM (purple) = Blomstrand; FGL (dark blue) = Fuglehuken; KRY (cyan) = Krykkjefjellet; OBS (green) = Observasjonsholmen; OSS (yellow) = Ossian Sarsfjellet) and glacier fronts (blue areas) are also shown. Maps were generated using R (version 4.0.2^42^; URL: https://www.R-project.org/).

This study was conducted in the Kongsfjorden region, Svalbard, during the chick-rearing period of 2017. The study area was delimited by a radius of 50 km from the centre of Kongsfjorden (Fig. 1). In total, 25 tidewater glacier fronts are present in the area and are used to a varying extent by kittiwakes breeding in the five studied colonies, i.e., Blomstrand (12.11° E, 78.99° N), Krykkjefjellet (12.18° E, 78.89° N), Observasjonsholmen (12.28° E, 78.93° N), Ossian Sarsjellet (12.44° E, 78.92° N), and one colony outside the fjord; Fuglehuken (10.47° E, 78.89° N) (Fig. 1). Following standardized procedures^35^, breeding pairs at each colony were counted once between 2011 and 2017 to assess colony size (Table 1). The kittiwake population of Svalbard was relatively stable from 2009 to 2019^36^, and hence we assumed that colony sizes assessed a few years prior to 2017 were representative of the colony sizes during our study.

**Table 1:** Number of black-legged kittiwake foraging trips recorded during the chick-rearing period in 2017 in five colonies located in the Kongsfjorden region, Svalbard.

Colony	Males	Females	Total	Colony size	Year
Blomstrand (BLM)	8 (2)	17 (3)	25 (5)	908	2011
Fuglehuken (FGL)	11 (8)	7 (5)	18 (13)	4286	2011
Krykkjefjellet (KRY)	13 (4)	23 (5)	36 (9)	318	2016
Observasjonsholmen (OBS)	35 (6)	48 (7)	83 (13)	141	2017
Ossian Sarsfjellet (OSS)	26 (5)	17 (3)	43 (8)	1936	2011
Total	93 (25)	112 (23)	205 (48)	7589	–

The number of individuals tracked in each colony is indicated in parentheses. The size (number of breeding pairs) of each colony and the year of the colony survey are also indicated.

### GPS tracking

In total, 48 birds were caught at the nest using a noose pole and were fitted with a GPS device (minimum and maximum dates of deployment: 7–28 July). Each individual was sexed, either by a molecular approach using DNA from blood or feather samples (69% of the individuals; see details in Harris et al.^37^) or by morphometry, using a cut-off value ($x$) of 90.5 mm on the birds’ head-bill length^38^ (31% of individuals: i.e., female ≤ $x$  > male). All birds had at least one chick hatched at the time of logger deployment. Three different types of GPS were deployed in random order (i.e., i-gotU GT-120, Mobile Action; CatLog Gen1, and CatLog Gen2). All GPSs were sealed in waterproof heat-shrink tubes, and their total mass ranged from 7.3–18.1 g ($\tilde{x } \pm SD$; 13.9 ± 2.9), representing 2–5% of the birds’ mass. Loggers were fitted to the birds’ back feathers with TESA tape. We found no indication that the mass of the GPS modified bird foraging behaviour (see details in Supplementary Information S1).

On average, the GPS devices deployed on birds were retrieved after 71.2 h (range = 23.5–160.5 h). Sampling intervals ranged from 2 to 10 min, and all tracks were subsampled to obtain a standard interval of 10 min between consecutive locations. Speed was filtered for a maximum of 80 km h^−1^ to remove aberrant locations^39,40^. We defined a trip as (1) all consecutive GPS positions located outside the colony area, which was defined as an area of 200-m radius from its centroid, and (2) being outside the colony’s area for a minimum of 50 min (thresholds based on sensitivity analyses, see Bertrand et al.^34^). To limit the number of non-foraging trips in the analyses, we excluded from subsequent analyses all tracks that had a proportion of locations over land equal to or greater than 50%, as kittiwakes can spend time bathing in freshwater^41^ and fetching nest materials on land. Moreover, only complete tracks were considered in the analyses (i.e. trips for which locations were registered until the bird returned to the colony). This choice did not bias samples towards shorter trips (i.e., we found no difference between the “complete” and “non-complete” groups in their maximum distances travelled per trip; Mann–Whitney U-test; *U* = 22 645, *p* = 0.94). In total, 205 foraging trips were used in this study (Table 1, see details in Supplementary Information S2), ranging from 1–10 per individual ($\overline{x } \pm SD$; 4.3 ± 2.7).

All bird captures and handling were performed following relevant guidelines and regulations regarding animal welfare in Norway (Forsøksdyrforvatningen tilsyns- og søknadssystem, *FOTS*). All field protocols were formerly approved by the ethics committees of the Governor of Svalbard (program for research 361) and the Norwegian Food Safety Authority (*Mattilsynet*; permits #6439, #8602, and #15503).

### Data analysis

All analyses were performed with R (version 4.0.2^42^).

#### Estimation of individual-level foraging ranges

We used an autocorrelated kernel density approach (AKDE^43,44^) to estimate each individual’s probability distribution (hereafter *utilization distribution; UD*) using the ctmm^45^ and ctmmweb^46^ packages. Contrary to conventional range estimates (e.g., Kernel Density Estimation (KDE), Minimum Convex Polygon), AKDE does not assume independency and identical distribution (IID) among GPS locations, and explicitly accounts for the temporal autocorrelation inherent to every trip while calculating an individual’s space use^47,48^. To reduce the weight of the locations at the colonies in individual UD calculations, we considered all consecutive trips from the same individual as continuous while removing all points located at the colony. All individual UDs were computed over a grid size of 500 m. In total, three different models were tested for each individual: (1) the IID, as assumed by conventional KDE, (2) the Ornstein–Uhlenbeck (OU: featuring positional autocorrelation timescale); and (3) the OU-Foraging processes (OUF: featuring both velocity and positional autocorrelation timescale)^49^. Model selection for each individual’s UD estimation was performed using the Akaike Information Criterion with correction for small sample sizes (AICc), and the model with the lowest AICc was selected^50,51^. We thereafter used the effective sample size (${\widehat{N}}_{area}$) as a proxy of an individual’s UD representativeness, which theoretically corresponds to the number of statistically independent positions represented by the tracks, corresponding roughly to the number of range crossings undergone by the animal during the tracking period^43,48^. A minimum ${\widehat{N}}_{area} \ge 4.5$ for each individual’s UD was considered as a minimal threshold^47,48^ (see details in Supplementary Information S3). Based on this threshold, 10 of the 13 individuals (i.e. ~ 77%) from Fuglehuken were filtered out. Finally, we assessed the representativeness of colony-level grouping as a function of sample sizes using the bootstrap approach described in Lascelles et al.^52^ and implemented via the track2kba package^53^ (see details in Supplementary Information S3).

#### Quantifying segregation level among colonies

Spatial segregation among colonies was evaluated using the calculated individual UDs and by extracting the Bhattacharyya coefficient (*BA*) calculated upon pairs of individual UDs^54–57^. This index relies on the probability density functions of the two UDs and does not require any subjective choice of specific quantile contours^54^. We used the inverse coefficient (i.e., $1-BA$; hereafter called the segregation index, *SI*), which ranged from 0 (complete overlap) to 1 (full segregation) and was computed using the ctmm package^45^ along the approximate debiasing correction for small sample size^54^. We used an analysis of similarities (ANOSIM) for testing the null hypothesis that the dissimilarity between colonies is equal to or lower than the dissimilarity among individuals within colonies^58^, or in other words, that segregation in space use between members of different colonies is equal to or weaker than that of members belonging to the same colony. For that purpose, we used the segregation matrix (i.e., pairwise segregation index (*SI*) among all individuals) as a response variable and the colony affiliation as a grouping factor. We considered that the hypothesis of segregation among colonies was supported if the dissimilarity among colonies was greater than the dissimilarity within colonies, using a nominal threshold of 0.05 along 999 permutations^59,60^. If the test was significant at the population-level (i.e., involving all colonies), we then tested segregation on a pairwise basis, this time applying ANOSIM analyses to each colony dyad. Since multiple testing was conducted, we controlled the false discovery rate using the Benjamini–Hochberg procedure^61^. Since the ANOSIM approach is sensitive to intragroup dispersion^58^, we also tested for the null hypothesis of homogeneity in group multivariate dispersion^62^. ANOSIM and the test for multivariate homogeneity were performed using the vegan package^63^.

#### Effect of distance on the probability to use glacier fronts

We used a generalized linear mixed model with a binomial distribution to test the prediction that kittiwakes were more likely to use glacier fronts located closer to their colony rather than those located farther away. We tested the prediction at two spatial scales (i.e., regional scale and fjord scale). First, to include all glaciers that birds could potentially use, we considered a radius of 50 km from the Kongsfjorden centre (corresponding to the distance of the most distant glacier front used by kittiwakes in our dataset). A total of 25 glacier fronts were located within that radius (i.e., regional scale; Fig. 1a). Second, we considered only the four colonies and 6 glacier fronts located within Kongsfjorden to confirm that the relationship observed at the regional scale was similar at a finer scale (i.e., fjord scale; Fig. 1b). We defined our response variable as binary: a value of 1 was assigned to a specific front if the birds used it during a given foraging trip, and the value 0 was assigned if they did not. We defined the front areas by creating a spatially-explicit buffer around the front line (radius = 400 m). Based on a previous study^34^, a glacier front was considered to be used when two consecutive locations (10-min interval) were within the buffer zone.

Distances between a colony and glacier’s front line centroid were calculated using the Great Circle distance via the sp package^64,65^ and were subsequently used as predictors of glacier front use. We also included individual sex as a predictor to control for potential variation between males and females^37,39,66^. The interaction between distance and sex was investigated to ensure that the space use patterns of colonies was not driven by a particular sex. Moreover, the colony was included as a fixed effect to account for the average difference in glacier front use among colonies. Significance of each predictor was evaluated by likelihood ratio tests for nested model, comparing the fits with (H_1_) and without (H_0_) the addition of the corresponding variable (nominal threshold of 0.05). For all models, we fitted the individual ID as random factors. We found no sign of overdispersion in the full models using the nonparametric dispersion test over quantile residuals (*p* = 0.87) available via the dharma package^67^.

#### Drivers of intercolony spatial segregation

Drivers of spatial segregation among colonies were investigated by means of a multiple regression on distance matrices (MRM^68^) using the ecodist package^69^. Colony size (Table 1), the distance separating colonies, and the dissimilarity in glacier front use were used as predictors and the segregation matrix as the response variable. We calculated the dissimilarity in glacier front use among individuals by using individual-level counts of use of each glacier (i.e., from the binary variable detailed in the previous section) and the pairwise individual similarity index from Bolnick et al*.*^70^:

$${H}_{ij}=\sum_{k}min\left({p}_{ik},{p}_{jk}\right)$$

where *p*_*ik*_ and *p*_*jk*_ represent the proportion of use of glacier *k* for the two selected birds (*i, j*). Since birds performed several trips during their tracking period, we first summed the counts by glacier during the trips of each individual before calculating the proportions. One individual (out of 13 birds) from Observasjonsholmen and all birds from Fuglehuken did not use any fronts during their tracking period and were thus removed from the analysis. For ease of interpretation, we subsequently used the inverse index, i.e., $DH=1-H$, hence generating a dissimilarity index. The dissimilarity index (*DH*) in glacier front use thus ranged from 0 (complete overlap in glacier front use) to 1 (complete segregation in glacier front use) and was calculated via the package rinsp^71^.

Due to its potential importance in modulating intraspecific competition for prey acquisition^11,13,72^, we controlled for colony size while investigating drivers of intercolonial segregation. We used the Manhattan distance, also known as the ${L}^{1}$ norm, to calculate the difference among the colony sizes. The Euclidean distance was chosen for calculating the distance separating colony centroid of each individual. Predictor significance was evaluated via permutations along 999 iterations and using the nominal threshold of 0.05.

## Results

**Figure 2:**
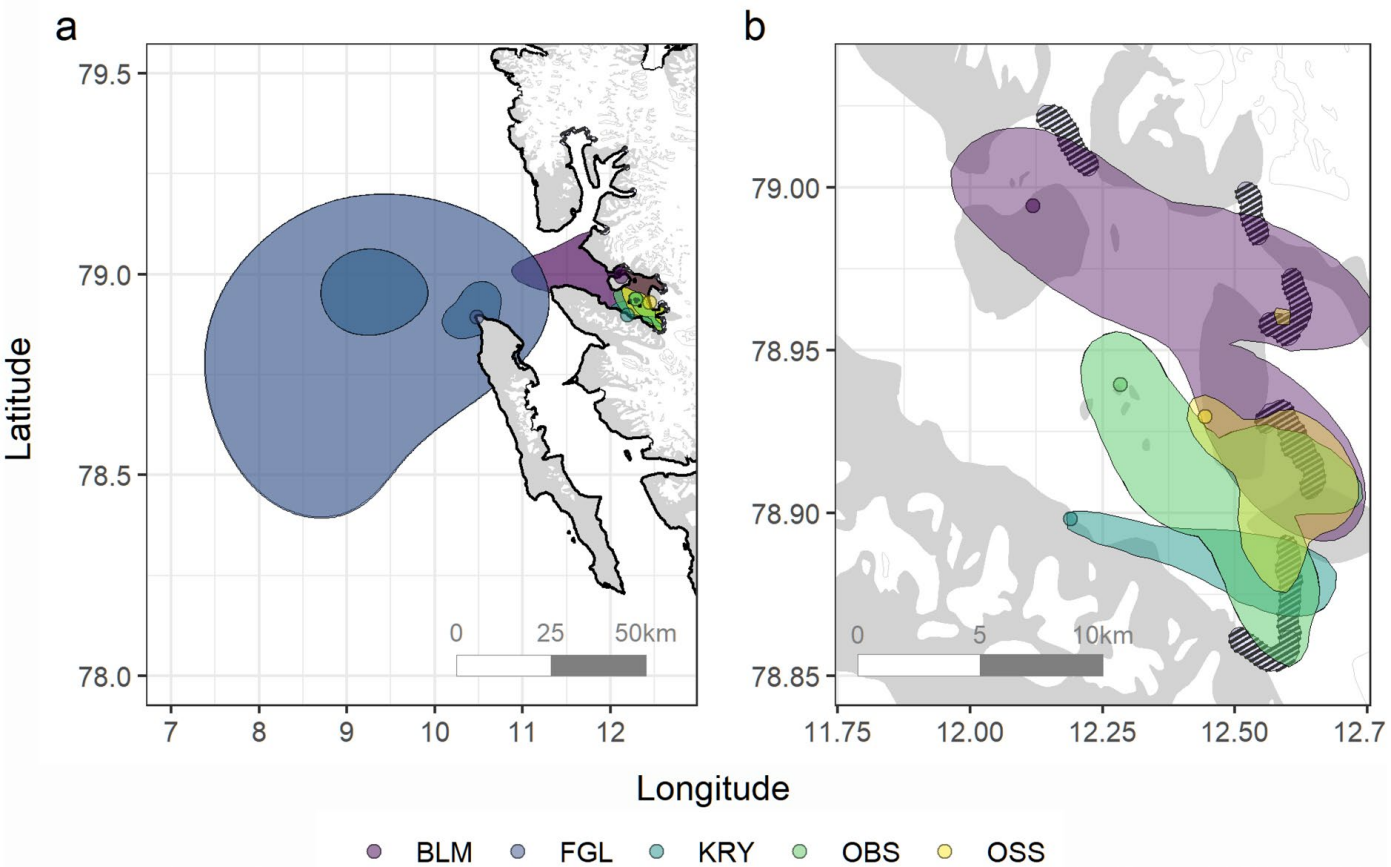
(**a**) Study area and the mean utilization distribution (UD) estimated for each kittiwake breeding colony (95% and 50% isopleths based on the individuals’ UD weighted by the number of trips sampled per individual; BLM (purple) = Blomstrand, FGL (dark blue) = Fuglehuken, KRY (cyan) = Krykkjefjellet, OBS (green) = Observasjonsholmen, OSS (yellow) = Ossian Sarsfjellet) and (**b**) core-ranges (50% isopleths) for the four colonies (circles) located within Kongsfjorden. Glacier fronts (crosshatched) are also shown. All birds were tracked during the chick-rearing period in 2017. Maps were generated using R (version 4.0.2^42^; URL: https://www.R-project.org/).

Space use varied substantially among the different kittiwake colonies. Birds breeding in the Fuglehuken colony showed no overlap with any glacier front, although the closest front was only 23 km away from the colony. Although all colonies have access to the pelagic zone, only birds from Fuglehuken relied exclusively on the pelagic environment to forage (Fig. 2a, see Supplementary Information S2 for raw tracks). Within Kongsfjorden, the core-use area derived for each colony (i.e., 50% isopleth) showed highly restricted and delimited space use, which always overlapped at least one glacier front (Fig. 2b). Representativeness analyses suggested that most of the intracolonial variation in space use was captured by colony UD estimates (range: 84–95%, see Supplementary Information S3 for details).

The global analysis of similarities (ANOSIM) indicated that the dissimilarity in space use was greater among colonies (average: 0.57; range: 0.39–0.98) than within colonies (average: 0.27; range: 0.17–0.50). This indicates that the overlap between foraging ranges was higher among birds from the same colony than among birds from different colonies (ANOSIM-*R* = 0.59, *p* = 0.001). The same pattern was observed when considering only the four colonies located within Kongsfjorden (ANOSIM-*R* = 0.50, *p* = 0.001). While showing no sign of heterogeneous dispersion among groups (*F*_*4,33*_ = 0.93, *p* = 0.457), the ANOSIM analysis conducted on each pair of colonies suggested that all colonies showed significant spatial segregation, except the Blomstrand (BLM)—Observasjonsholmen (OBS) dyad (Table 2). Although the core-use area of these two colonies showed little overlap, it suggests that some birds breeding in these colonies shared similar foraging space (see raw tracks distribution in Supplementary Information S2).

**Table 2:** Results of pairwise multilevel comparison of segregation among the five colonies (BLM = Blomstrand; FGL = Fuglehuken; KRY = Krykkjefjellet; OBS = Observasjonsholmen; OSS = Ossian Sarsfjellet) in Kongsfjorden using ANOSIM (*R*; below diagonal) and the distance (km) separating each pair of colonies (upper diagonal).

	BLM	FGL	KRY	OBS	OSS
BLM	–	36.87	10.84	7.07	10.04
FGL	*R* = 1.00; ***p* = 0.024**	–	36.78	39.07	42.40
KRY	*R* = 0.95; ***p* = 0.003**	*R* = 1.00; ***p* = 0.013**	–	5.02	6.50
OBS	*R* = 0.23; *p* = 0.059	*R* = 1.00; ***p* = 0.005**	*R* = 0.48; ***p* = 0.003**	–	3.62
OSS	*R* = 0.41; ***p* = 0.013**	*R* = 1.00; ***p* = 0.013**	*R* = 0.88; ***p* = 0.003**	*R* = 0.25; ***p* = 0.014**	–

Significance (shown in bold) was evaluated along 999 permutations using the false-discovery rate correction for multiple testing (see “Methods” section).

**Figure 3:**
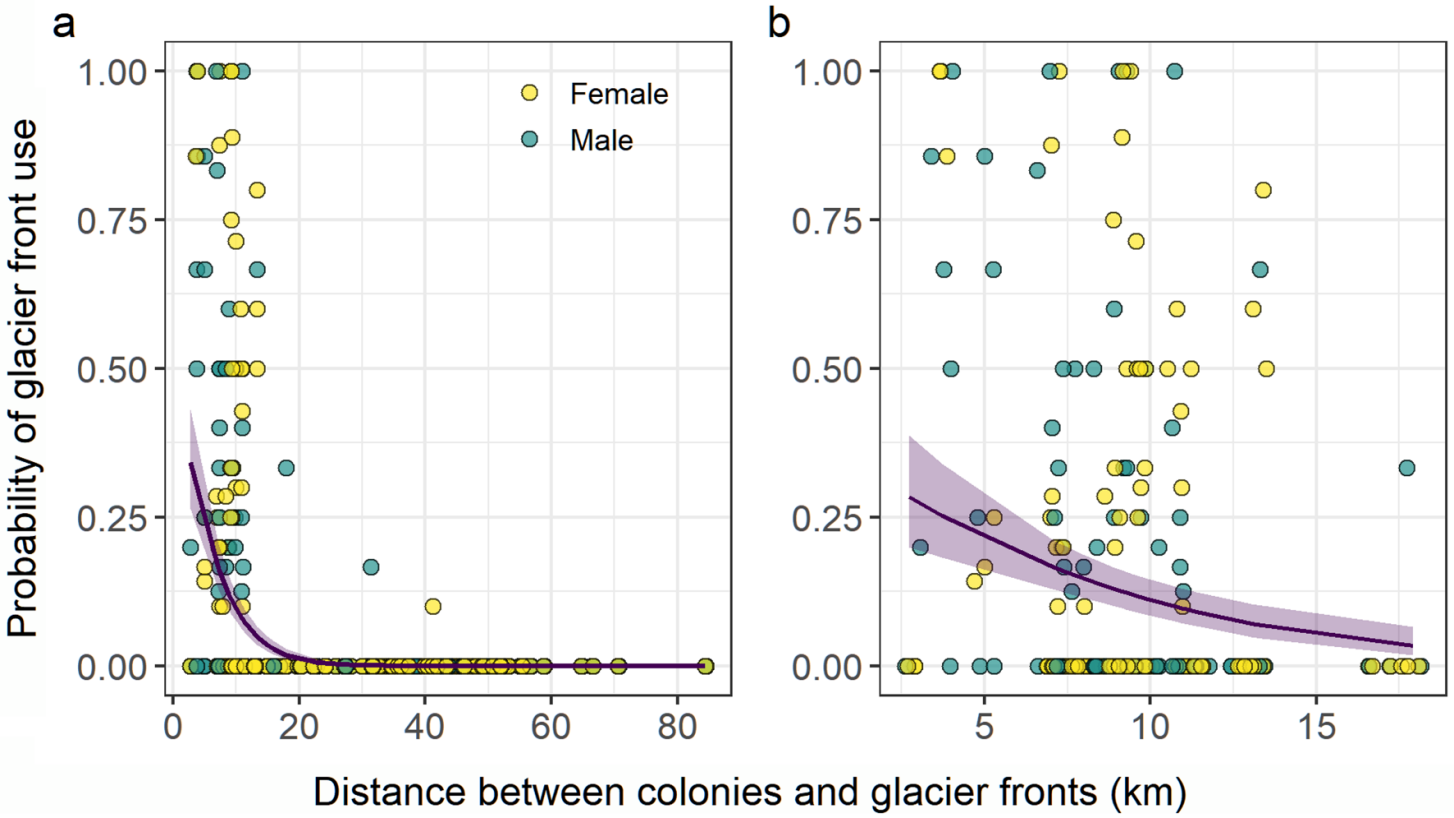
Relationship between black-legged kittiwake use of glacier fronts as a function of the distance separating their colonies to a given front at two different scales; (**a**) regional scale; involving 5 colonies and 25 glacier fronts (effect of distance; estimate =  − 0.22, 95% CI =  − 0.26: − 0.18) and (**b**) fjord scale; involving 4 colonies and 6 glacier fronts (effect of distance; estimate =  − 0.16, 95% CI =  − 0.22: − 0.09). For the ease of representation, points represent individual males and females average use (circles: yellow = female; green = male) of glacier fronts. Solid curves and shaded areas are the regression line and associated 95% confidence interval (back-transformed) estimated from generalized linear mixed models, using the glacier front use as response variable, the distance, sex and colony as additive fixed predictors and the bird ID as random factor.

Glacier front use varied substantially for birds breeding in various neighbouring colonies located within Kongsfjorden (see details in Supplementary Information S4). The use of glacier fronts decreased significantly with the distance separating them to a given colony at the two different spatial scales used for the analysis (Table 3, Fig. 3). A threshold around 18 km was also observed at the regional scale, which corresponded to the maximum distance separating a colony and a front located within Kongsfjorden (Fig. 3a). This indicates that kittiwakes breeding in Kongsfjorden essentially used glacier fronts located inside the fjord. A similar decaying relationship was observed at the fjord scale when considering only colonies and glacier fronts occurring within Kongsfjorden (Fig. 3b). All models showed no effect of individual sex (Table 3).

**Table 3:** Binomial generalized linear mixed models testing the effect of colony, distance, sex (female as reference level) and their interaction on the use of glacier fronts by black-legged kittiwakes at two different scales: (1) *Regional scale*, based on all glacier fronts occurring in a radius of 50 km from Kongsfjorden’s centroid and (2) *Fjord scale*, based on all glacier fronts occurring in Kongsfjorden, Svalbard. Colony was included as a fixed effect to account for their average difference in glacier front use.

Effect	Distance	Sex	Distance × sex	Colony	*cR*^2^
Model H_0_	Sex + colony	Distance + colony	Distance + sex + colony	Distance + sex	–
Model H_1_	Distance + sex + colony	Distance + sex + colony	Distance × sex + colony	Distance + sex + colony	–
*Regional scale*	${{\varvec{X}}}_{\mathbf{1}}^{\mathbf{2}}$**= 549.78; *p* < 0.001**	${X}_{1}^{2}$ = 0.31; *p* = 0.581	${X}_{1}^{2}$ = 1.72; *p* = 0.190	${X}_{4}^{2}$ = 7.96; *p* = 0.093	0.59
*Fjord scale*	${{\varvec{X}}}_{\mathbf{1}}^{\mathbf{2}}$** = 24.58; *p* < 0.001**	${X}_{1}^{2}$= 0.31; *p* = 0.578	${X}_{1}^{2}$ = 3.12; *p* = 0.077	${{\varvec{X}}}_{\mathbf{3}}^{\mathbf{2}}$** = 7.86; *p* = 0.049**	0.04

The individual ID was fitted as random effects. Likelihood ratio Chi-squared statistic and associated *p* values are given, with models specifying the null (H_0_) and alternative (H_1_) hypotheses specified for each test. *cR*^2^ is the conditional *R*^2^ (i.e., for both fixed and random effects*)* for the model using the distance, sex and colony as additive fixed predictors.

Significance (shown in bold) based on the nominal threshold of 0.05

**Figure 4:**
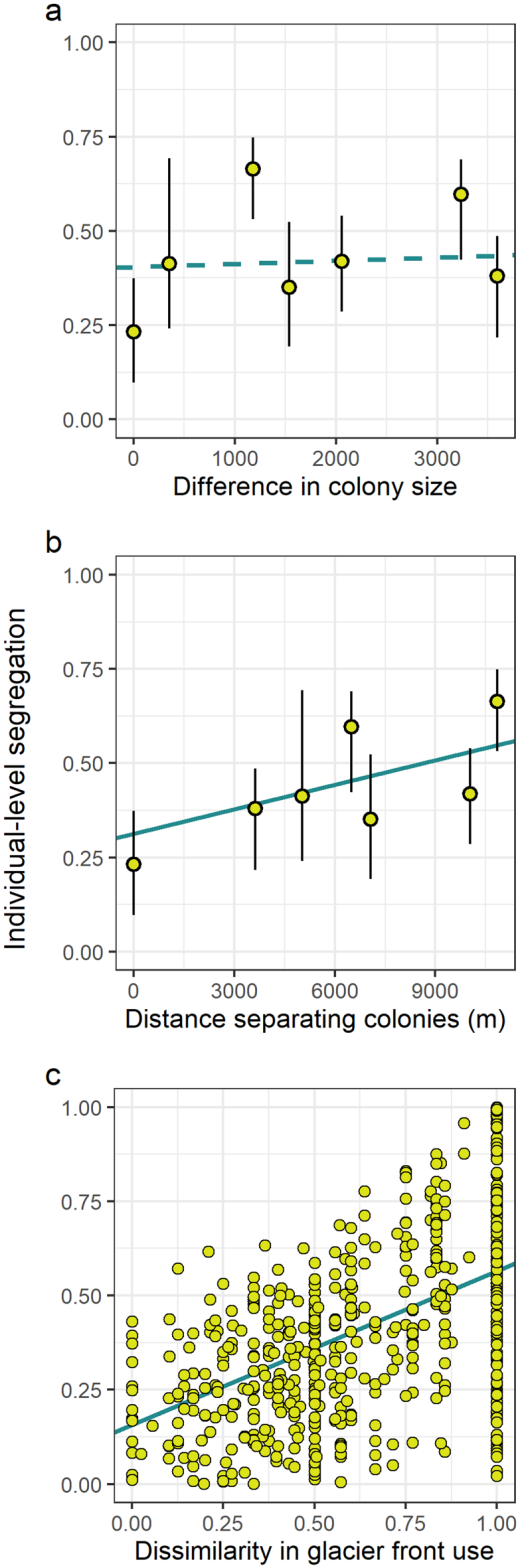
Spatial segregation level (i.e., 1 − Bhattacharyya coefficient) of breeding kittiwake foraging ranges as a function of (**a**) the difference in colony size (i.e., Manhattan distance), (**b**) the distance separating colonies, and (**c**) the dissimilarity in glacier front use between each individual dyad. Solid and dashed lines depict, respectively, the significant and non-significant partial slopes of the multiple regression on distance matrices. Intercepts of each slope have been adjusted for illustration using as reference the mean of the remaining covariates. Symbols represent the median and associated 25th and 75th percentiles in panels (**a**) and (**b**).

Spatial segregation in foraging ranges of breeding kittiwakes was positively correlated to the distance separating their colonies (estimate = 2.17 × 10^−5^, *p* = 0.001) and the level of dissimilarity in glacier front use (*DH*; estimate = 0.41, *p* = 0.001; Fig. 4). Surprisingly, however, segregation was not related to the relative size of the colonies (estimate = 8.31 × 10^–6^, *p* = 0.44; Fig. 4). Overall, the distance separating colonies and the dissimilarity in glacier front use explained about 41% of the spatial segregation in foraging ranges (*F* = 191.10, *p* = 0.001).

## Discussion

The investigation of marine predator movements is central to understanding how individuals respond to seascape dynamics and structures^11,73,74^. In this study, we tested the hypothesis that spatially-predictable seascape elements like tidewater glacier fronts can influence kittiwake foraging patterns and drive spatial segregation among adjacent colonies. Although chick-rearing kittiwakes can feed up to ca. 280 km away from their colony (see also Christensen-Dalsgaard et al.^40^), individuals in Kongsfjorden were more likely to use glacier fronts located closer to their colony and rarely used glacier fronts located > 18 km away. As predicted, the dissimilarity in the use of glacier fronts partly explained the spatial segregation observed between adjacent kittiwake colonies. Overall, our results illustrate how spatially-predictable foraging patches like glacier fronts can modulate the magnitude of intercolonial segregation in central-place foragers.

Optimal foraging theory predicts that central-place foragers should spend more time in highly profitable foraging patches located close to their breeding colony^75,76^. When two neighbouring colonies are separated by less than the distance covered by their members’ foraging range, patch predictability and distribution between the colonies could strongly affect the behaviour of foraging individuals and determine their level of spatial segregation^11,17^. As predicted, the spatial distribution of tidewater glacier fronts, which can offer relatively predictable foraging patches to various marine predators^22,30,33^, seems to partly drive consumer movements and promotes small-scale intercolonial segregation in our study system. However, glacier fronts might not offer equal foraging opportunity to breeding birds^33^, and the relatively low explanatory power of the distance between colony and glacier fronts on the probability of front use highlights the needs for further research on the relative profitability of these foraging patches.

Tidewater glacier fronts are often defined as foraging hotspots for various Arctic predators^22,26,31^. Subglacial plumes constitute major components of the mechanism modulating glacier front profitability for marine predators^28,30^. Originating from the subglacial meltwater discharge, the buoyant meltwater plume entrains water masses from intermediate depths and transports prey to the surface while making them accessible to predators^22,28,30^. A plume’s entrainment capacity relies on both the discharge velocity and the depth at which the subglacial plume originates^29,77^, which are both known to vary among glacier fronts in Kongsfjorden^33,78,79^. Glacier fronts in Kongsfjorden thus likely offer variable entrainment capacity and level of predictability. Moreover, the relative profitability of a given glacier front can vary depending on the environmental conditions^30–34^. Integrating such spatiotemporal variation in the profitability of glacier fronts should strongly help understanding their impacts on the spatial distribution of Arctic marine predators^78,80^. Along deglaciation, however, retreat of glacier termini above the sea-level in Kongsfjorden could ultimately reduce foraging opportunities in the fjord and modify the spatial distribution of seabirds by increasing their use of more distant, pelagic feeding areas as seen in kittiwakes breeding outside the fjord (see Fig. 2).

The spatial distribution of marine predators is known to be dependent on the distribution and patchiness of prey, which is strongly affected by physical forcings that promote their aggregations^73,81^. Eddies^66,82,83^ and fronts^83–87^ are oceanographic features that are associated with important foraging habitats for seabirds, by either enhancing primary production through the transport of nutrients or by the advection of biomass^88^. Ultimately, the spatiotemporal variability of these physical processes can modulate the level of predictability of prey aggregations in the seascape, which in turn affects the profitability of these patches to marine predators^19,20,74^. In parallel, foraging patches like glacier fronts that are predictable in space, and to some extent in time during a given summer^89^, are likely to promote foraging site fidelity^20,90^ and aggregations in seabirds^91,92^. If knowledge of locations of prey and/or foraging grounds is transferable to conspecifics at the colony level through the use of public information (e.g., bearings of departing/returning foraging birds^93,94^), then a cultural foraging pattern could arise and enhance intercolonial difference in space use^5,11,15^.

Our results add to a growing body of research indicating that segregation level in marine bird colonies is affected by seascape structures^11,17,18,95^. In a study conducted on Cory’s shearwater (*Calonectris borealis*), Ramos et al.^17^ showed that birds from different colonies tended to segregate in most of their foraging ranges, but overlapped largely in areas characterized by one predictable but distant foraging patch (i.e., a large upwelling area^17^). Similarly, Dean et al.^18^ showed that colonies of Manx shearwater (*Puffinus puffinus*) were more likely to segregate around their colony while overlapping at a distant, productive tidal front system. Taken altogether, these results indicate that the distribution of high-profitability foraging hotspots can have important effects on the spatial segregation of colonial breeding predators^11,17^. The varying level of segregation over such foraging patches (from overlap to segregation) likely reflects the distribution of individuals maximizing their net energy gain among all patches available in the seascape^75,96^. Further research is warranted to understand the complex relationship between central-place forager behaviors, space use and seascape heterogeneity^20,74,88^.

## Supplementary Information

Supplementary Information.

## Data Availability

The dataset that supports current findings is available from the dryad digital repository: 10.5061/dryad.bnzs7h4c1. R codes can be accessed from the github repository: https://github.com/PhilBertrand/GF_segregation.
