# Supplementary material for: Fine-scale spatial segregation in a pelagic seabird driven by differential use of tidewater glacier fronts
Source: Sci Rep. 2021 Nov 11;11:22109. doi: 10.1038/s41598-021-01404-1 (PMC8586018; doi:10.1038/s41598-021-01404-1)
Supplement: Supplementary file 1 — Supplementary Information. [file 41598_2021_1404_MOESM1_ESM.pdf]

Supplementary Information

Fine-scale spatial segregation in a pelagic seabird driven by differential use of tidewater glacier fronts

Philip Bertrand, Joël Bêty, Nigel Gilles Yoccoz, Marie-Josée Fortin, Hallvard Strøm, Harald Steen, Jack Kohler, Stephanie M. Harris, Samantha C. Patrick, Olivier Chastel, Pierre Blévin, Haakon Hop, Geir Moholdt, Joséphine Maton, & Sébastien Descamps

Supplementary Information S1 2
Supplementary Information S2 3
Supplementary Information S3 4
Supplementary Information S4 7
References 8

Supplementary Information S1

Effect of GPS on foraging behaviour

The GPSs were distributed in random order in the chick-rearing period of 2017. This design lets us explore the effect of a GPS's weight on kittiwake foraging behaviour. A total of 205 trips from 48 individuals were collected.

We tested whether the GPS's relative mass (% of bird mass) was modulating the distance travelled by the birds in their foraging trip. A positive relationship would indicate that birds would increase the distance flown with a heavier GPS. A negative relationship would imply that the bird remains close to the colony (and thus the fjord and associated glacier fronts) with heavier devices.

We used a mixed model with a Gaussian error structure, using the maximum distance flown per trip (km) as a function of the GPS's relative mass (%). Since the distribution of maximum distances was highly skewed, we log-transformed the variable to meet the assumption of homoscedasticity. We furthermore controlled for the sex of the bird and used the bird ID as a random factor. Models were compared using Akaike's information criterion (AIC). We used the R package NLME (Pinheiro et al. 2020) to perform the regressions and the package AICCMODAVG (Mazerolle 2020) for the AIC calculation.

Table S1.1. Estimates and approximate confidence intervals for the mixed models testing the effect of GPS relative mass (%) and sex on the (log-transformed) maximum distance flown by kittiwakes per trip. AIC estimates are based on models using the maximum likelihood (ML) procedure and model estimates by the restricted maximum likelihood (REML). 95% confidence intervals (in parentheses) for all estimates are also shown.

Model	AIC	ΔAIC	sex (male)	GPS mass	sex * GPS mass
null	505.76	0.00	-	-	-
GPS	506.98	1.22	-	2.6 (-18.7:24.0)	-
sex (male)	507.66	1.91	-10.6 (-43.2:22.0)	-	-
sex + GPS	508.82	3.07	-10.4 (-43.5:22.8)	2.1 (-19.4:23.7)	-
sex * GPS	509.93	4.17	-65.9 (-223.7:91.9)	-5.3 (-35.4:24.7)	15.7 (-27.9:59.2)

These results suggest that the GPS's relative mass is not modulating the distance flown by the birds in their foraging trips (95% confidence interval of the predictor's estimate overlaps zero). It also indicates that the space used by the birds is likely not driven by the tracking device's weight.

Supplementary Information S2

Distribution of raw tracks for the five monitored colonies

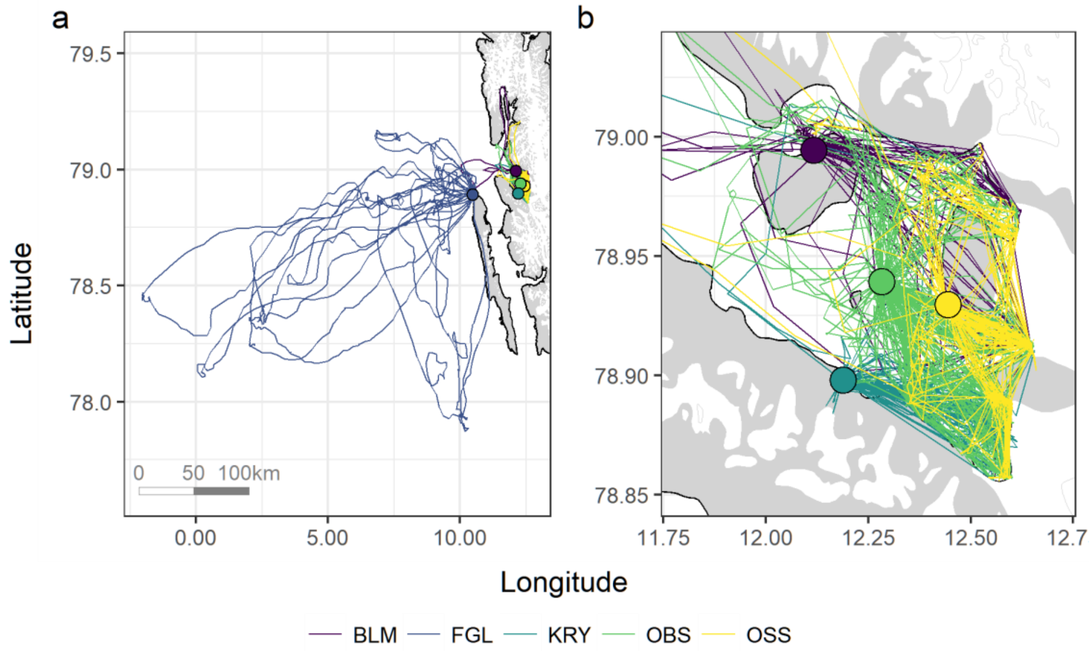

Figure S2.1. Delineated tracks (lines) mapped from a) the whole study area and b) Kongsfjorden area of the five colonies (circles) monitored during chick rearing in 2017 *i.e.* Blomstrand (BLM; purple), Fuglehuken (FGL; dark blue), Krykkjefjellet (KRY; cyan), Observasjonsholmen (OBS; green) and Ossian Sarsfjellet (OSS; yellow).

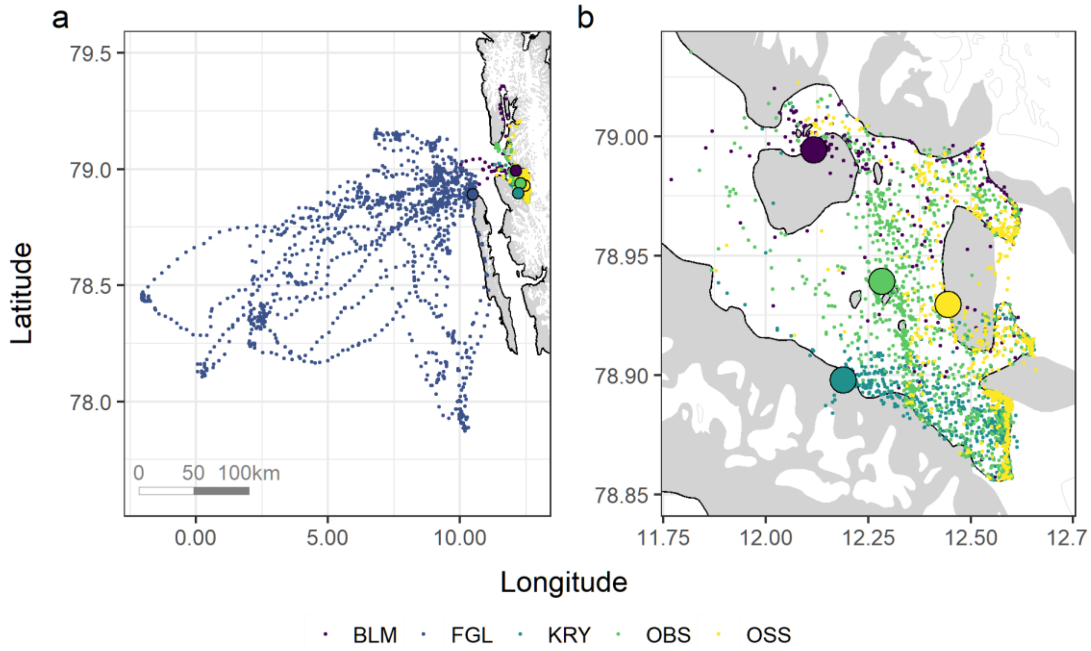

Figure S2.2. Distributions of GPS locations (small circles) from a) the whole study area and b) Kongsfjorden area of the five colonies (large circles) monitored during chick rearing in 2017 *i.e.* Blomstrand (BLM; purple), Fuglehuken (FGL; dark blue), Krykkjefjellet (KRY; cyan), Observasjonsholmen (OBS; green) and Ossian Sarsfjellet (OSS; yellow).

Supplementary Information S3

Home range sampling details using the autocorrelated kernel density estimator (AKDE)

In total, three different models have been tested for each individual, with both their isotropic (*i.e.*, movement process independent of directions) and anisotropic (*i.e.*, movement process dependent of directions) versions: (1) the IID, as assumed by conventional KDE, (2) the Ornstein-Uhlenbeck (OU: featuring positional autocorrelation timescale), and (3) the OU-Foraging processes (OUF: featuring both velocity and positional autocorrelation timescale; OUf: if velocity and positional autocorrelation timescale are considered identical). The difference between models are shown in fig. S3.1. Table S3.1 shows how many individuals per colony have been fitted with these models after model selection (*i.e.*, based on Akaike's Information Criterion with correction for small sample sizes (AICc)).

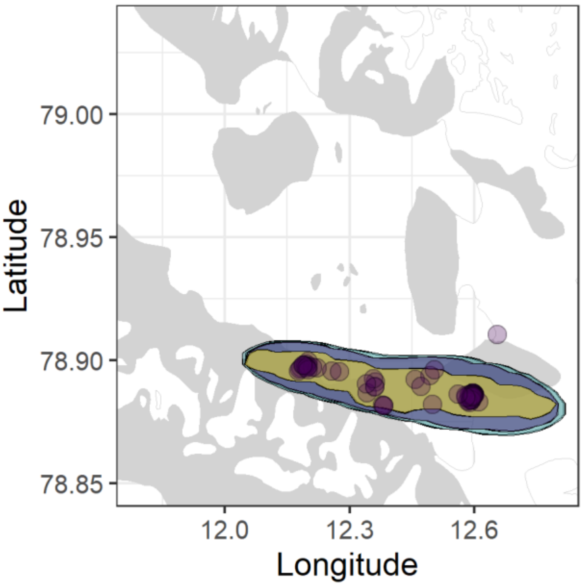

Figure S3.1. Three models outputs for one chick-rearing individual from Krykkjefjellet in 2017. The green area corresponds to the OU model, the blue area to the OUF model and the yellow area to the IID model. GPS locations are also displayed (see Methods for details).

Table S3.1. Model type and associated number of chick-rearing individuals by colonies in Kongsfjorden area in 2017.

Model	OSS	OBS	KRY	BLM	FGL
OU anisotropic	-	1	3	-	-
OUF anisotropic	6	9	4	3	-
OUF isotropic	1	2	1	1	1
OUf anisotropic	1	1	1	1	-
OUf isotropic	-	-	-	-	2
Total	8	13	9	5	3

Due to the low number of individuals sampled in some of the colonies (Table 1), we assessed the representativeness of each colony-level UD estimates using the bootstrap approach described in

Lascelles et al. (2016) and implemented via the TRACK2KBA package (Beal et al. 2020). Briefly, the approach consists of monitoring core range (*i.e.*, 50% isopleth) distribution changes as a function of each colony's sample size. This was achieved by calculating a colony-level inclusion index, which is computed using an iterative process: (1) by randomly selecting a subset of individuals from a colony ("selected individuals"), (2) averaging their individuals' UD, and (3) calculating the proportion of the trips of the "non-selected" individuals (*i.e.*, inclusion index) that are overlapping the "selected individuals" mean UD. In other words, this inclusion index summarizes to which extent our sample size for each colony accounts for the variability of individual space use. We calculated the inclusion indices for every sample size (*i.e.*, $n_i \ldots n_{i-1}$) along 999 iterations. From the bootstrapped dataset, we used a nonlinear model fitted by least squares using the inclusion indices as response and the sample sizes as a predictor to estimate the horizontal asymptote of the function. The representativeness of each colony was then calculated as the average inclusion index (estimated via the nonlinear model) obtained from the maximum sample size (*i.e.,* n_{i-1}) divided by the asymptote. All colonies tracked in Kongsfjorden (Fuglehuken was not evaluated due to the very low sample size) had 84-95% of their core ranges covered by their relative sample sizes of n_{i-1} (Table S3.2, Fig. S3.1).

Table S3.2. Colony and respective sample size (number of individuals), the median of the effective sample size (number of points for representing IID), the effective sample size for the colony-level (weighted average) UD, associated asymptote, and % represented by the data of the median core area (50%) (see Methods for details).

Colony	*n*	Median (range) $\hat{N}_{area}$	Weight. Ave. $\hat{N}_{area}$	Asymptote	Represent. (%)
OSS	8	22.62 (12.20-39.06)	390.99	0.75	84.37
OBS	13	23.27 (4.66-43.63)	603.62	0.68	87.26
KRY	9	11.48 (6.82-29.37)	230.45	0.70	94.89
BLM	5	13.53 (5.31-32.02)	160.86	0.66	93.10
FLG	3	5.38 (5.13-5.83)	32.67	NA	NA

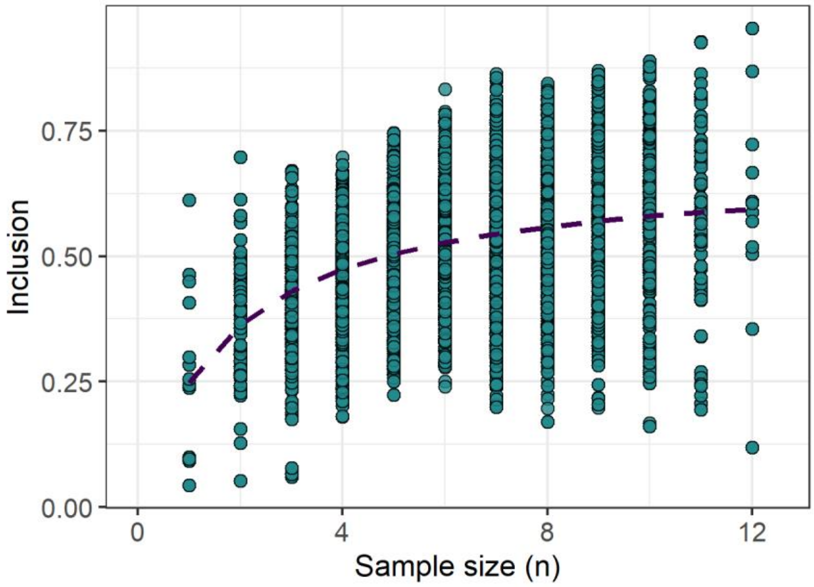

Figure S3.1. Nonlinear model fitted by least squares using the inclusion index as a function of the sample size and associated horizontal asymptote for the Observasjonsholmen colony in Kongsfjorden.

Supplementary Information S4

General proportions of glacier front use

For comparison between colonies, we calculated the seasonal average glacier front use per colony using the following steps: (1) we calculated the proportion of trips that used at least one glacier front for each individual tracked in the five colonies, (2) we calculated the colony's average use of glacier fronts using these individual proportions, but weighted values using the number of trips recorded per individual (Fig. S4.1). Averages' confidence intervals (95%) were computed by bootstrapping the individuals' proportion and associated weight along 999 iterations using the BOOT package (Canty & Ripley 2020). We reported the 2.5% and 97.5% percentiles.

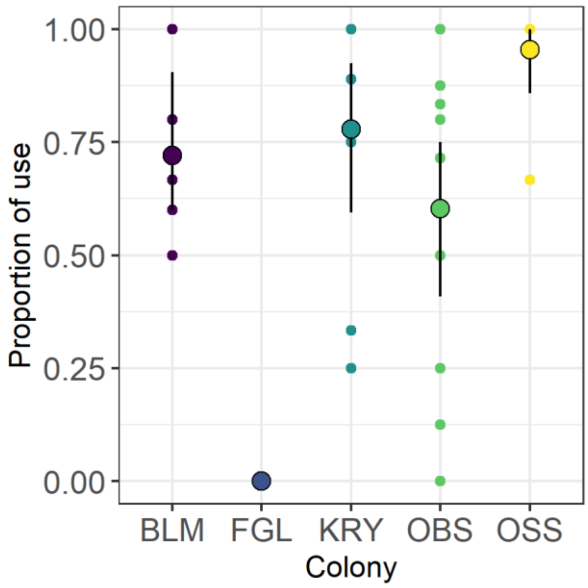

Figure S4.1. The proportion of use (trip binary use averaged per individual, giving a proportion per individual) of glacier fronts in Kongsfjorden and Krossfjorden for the five colonies investigated (*i.e.*, BLM = Blomstrand, FGL = Fuglehuken, KRY = Krykkjefjellet, OBS = Observasjonsholmen and OSS = Ossian Sarsfjellet).

References

Beal M, Oppel S, Handley J, Pearmain L, Morera-Pujol V, Miller M, Taylor P, Lascelles B, Dias M (2020) BirdLifeInternational/track2kba: First Release (Version 0.5.0). Zenodo. http://doi.org/10.5281/zenodo.3823902.

Canty A, Ripley B (2020) Boot: Bootstrap R (S-Plus) Functions. R package version 1.3-25.

Mazerolle MJ (2020) AICcmodavg: Model selection and multimodel inference based on (Q)AIC(c). R package version 2.3-1. URL: https://cran.r-project.org/package=AICcmodavg.

Pinheiro J, Bates D, DebRoy S, Deepayan S, Team RC (2020) Nlme: Linear and Nonlinear Mixed Effects Models. R package version 3.1-149. URL: https://cran.r-project.org/package=nlme.
